# Supplementary material for: Estimating the impact of new high seas activities on the environment: the effects of ocean-surface macroplastic removal on sea surface ecosystems
Source: PeerJ. 2023 Apr 27;11:e15021. doi: 10.7717/peerj.15021 (PMC10149055; doi:10.7717/peerj.15021)
Supplement: Supplemental Information 2 [file peerj-11-15021-s002.docx]

# Supplementary Material

Estimating the impact of new high seas activities on the environment: The effects of ocean-surface macroplastic removal on sea surface ecosystems

Matthew Spencer^1^, Fiona E Culhane^1, 2^, Fiona Chong^3^, ^4^, Megan O Powell^5^ Rozemarijn J Roland Holst^6^, and Rebecca R Helm^7 *^

1 School of Environmental Sciences, University of Liverpool, Liverpool, United Kingdom

2 School of Biological and Marine Sciences, University of Plymouth, Plymouth, United Kingdom

3 Energy and Environment Institute, University of Hull, Hull, United Kingdom

4 Biological and Marine Sciences, University of Hull, Hull, United Kingdom

5 University of North Carolina at Asheville, Asheville, NC, United States of America

6 Durham Law School, Durham University, Durham, United Kingdom

7 Earth Commons Institute, Georgetown University, Washington, DC, United States of America

Corresponding author:
Rebecca Helm
Email address: rh1203@georgetown.edu

**Table S1** Ecosystem services (ecological and societal benefits of neuston) provided by the zooneuston and macroalgae found on the ocean surface i.e. the neuston. There are three types of service: Provisioning (P), Regulation and maintenance (R&M), and Cultural (C). Definitions of the services are derived from (Culhane et al., 2019). Links can be direct or indirect (see methods in main text). Only service supplied by the neuston are shown^1^.

| **Ecosystem Service** | **Description** | **Relevant community property for service supply (Biomass/Abundance/Species richness/Food web)** | **Criteria for inclusion** |
| --- | --- | --- | --- |
| **P: Seafood from wild plants and algae**  Indirect link *Macroalgae* | Several *Sargassum* seaweed species are cultivated and widely consumed in Asia, such as *S. fusiforme* (Xie et al., 2013). The two species of truly pelagic *Sargassum* that make up the Sargasso Sea: *S. natans* and *S. fluitans* are not currently cultivated, but there is evidence of people in the United States, e.g. in the state of Florida (Deane, 2013), and in the Caribbean (Caribbean Alliance for Sustainable Tourism, 2015) harvesting and eating these pelagic *Sargassum* when they get washed ashore. The presence of pelagic *Sargassum* in coastal areas and shores is becoming increasingly predominant as a result of global climate change and human activities (Louime et al., 2017). | A loss of abundance and biomass are likely to impact the supply of this service. This service is indirect because the service is directly supplied in different habitats (e.g. coastal, freshwater) and at different life stages of the relevant species, and not directly in the open ocean. | Actual use |
| **P: Seafood from wild animals**  Indirect link *Zooneuston* | Parts of the neuston consist of juvenile fish and fish eggs of commercial species that go on to supply the seafood service at different stages of their lives and in different habitats. A number of commercial species found in the neuston include: snapper (Lyczkowski-Shultz and Hanisko, 2007), Coho salmon (Pool et al., 2012) and eels (McCleave et al., 1987). | A loss of abundance and biomass are likely to impact the supply of this service. This service is indirect because the service is directly supplied in different habitats (e.g. coastal, freshwater) and at different life stages of the relevant species, and not directly in the open ocean. | Actual use |
| **P: Raw materials**  Indirect link  *Macroalgae, Zooneuston* | Raw materials are supplied by neuston through directly harvesting and extracting the desired properties. Ornamental objects can be derived from neuston that washes ashore and is collected by beach combers (Viney, 2014). *Sargassum* from the open ocean washed ashore is harvested and processed as a construction material in Mexico (Yucatan Times, 2018).  Fish oil supplements production uses parts from fish that are especially rich in the beneficial oils known as omega-3 fatty acids. These oily fish including mackerel, herring, salmon and cod (Kris-Etherton et al., 2002) spend part of their life cycle in the neuston (Hempel and Weikert, 1972, NOAA Fisheries, 2020). | Loss of biomass would impact on the *Raw materials* service, where the benefits rely on the potential to harvest in sufficient quantities. This service is indirect because the service is directly supplied in different habitats (e.g. coastal) and at different life stages of the relevant species. There is no clear evidence that harvesting of raw materials occurs directly in the open ocean. | Actual use |
| **P: Materials for agriculture and aquaculture**  Indirect link  *Macroalgae, Zooneuston* | When oceanic *Sargassum* gets washed ashore, communities in Barbados have harvested, processed and sold this resource as an organic mulch ‘Ocean Surf Organic Local Garden Mulch’ (Atwell, 2011, Caribbean Alliance for Sustainable Tourism, 2015) to add to soil to suppress weeds and control moisture evaporation. Sargassum is also processed and sold across North America and some Caribbean countries as a liquid fertiliser by Algas Organics, a company in St. Lucia (Ewing-Chow, 2019).  The Atlantic Menhaden (*Brevoortia tyrannus*), a species of herring with an egg and larval stage in the open ocean, has been used since potentially well before the 1600s as a fertiliser by Native Americans and European settlers (Ceci, 1975). Today, the fish is processed into fish meal, fish oil and fish solubles, where parts are used to create high protein feeds for livestock; or to fortify fish meal to increase nutrition in the aquaculture industry (NOAA Fisheries, 2020). | Loss of biomass would impact on this service, where the benefits rely on the potential to harvest in sufficient quantities. This service is indirect because the service is directly supplied in different habitats (e.g. coastal) and at different life stages of the relevant species. There is no clear evidence that harvesting of raw materials occurs directly in the open ocean. | Actual use |
| **P: Genetic materials**  Direct link  *Macroalgae, Zooneuston* | The neuston has a number of potential applications across a range of industries, through isolating properties that can later be reproduced under artificial conditions (*Genetic materials*). The blue pigment characteristic of many of the neuston is a type of astaxanthin protein (Zagalsky and Herring, 1977); this protein has a multitude of uses for aquaculture (for health and marketability of fish), and human health benefits when taken as a supplement (Higuera-Ciapara et al., 2006). Extracts from neuston animals has also been found to have antioxidant activity (Brekhman et al., 1981). In addition, the microbiome associated with neuston is known to have potential uses for biotechnology, such as for medical and industrial uses. For example, the bacteria *Alteromonas* sp., which is associated with neuston, has antibiotic properties against pathogens of fish, molluscs and crustaceans, thus has potential applications in the aquaculture sector (León et al., 2005). | Loss of species richness would impact on the *Genetic materials* service, where the benefits rely on the potential to bioprospect and extract properties once (that can then be re-produced in artificial conditions), as greater species diversity is likely to lead to a greater range of properties that can have potentially important uses. Losses to species that we do not know of yet, or that we know little about is particularly pertinent to this service, as we will lose any future potential genetic or raw material value. | Potential use |
| **R&M: Waste and toxicant treatment**  Direct link  *Macroalgae, Zooneuston* | The suspension, boring, detritus and scavenging modes of feeding amongst the neuston (Thiel and Gutow, 2005) mean that they have good capacity to breakdown, remove and bioremediate organic and other waste from the surface of the ocean. In addition, macroalgae can treat waste through phytodegradation (Susarla et al., 2002). | Losses to biomass and abundance are likely to be the most important factors in impacting the supply of this service. | Function |
| **R&M: Waste and toxicant removal and storage**  Direct link  *Macroalgae, Zooneuston* | All invertebrates can store hazardous substances in their body tissues (Martincié et al., 1984). Neuston are known to store mercury (Buckman et al., 2018), lead, cadmium and copper (Schulz-Baldes, 1992) in their tissues. This thus removes these substances, which can be hazardous at higher than natural concentrations, from circulation in the ecosystem, while they are alive. As a proportion of the neuston will die, sink and be integrated in the sediment, this can also remove these substances over a longer timescale. Macroalgae, such as sargassum, are also well known to sequester and store toxicants and wastes in their tissues (Sung et al., 2013). | Losses to biomass and abundance are likely to be the most important factors in impacting the supply of this service. | Function |
| **R&M: Mediation of smell/ visual impacts**  Direct link  *Macroalgae, Zooneuston* | The varied feeding modes of neuston (Thiel and Gutow, 2005) are likely to remove wastes and regulate conditions at the ocean surface that could lead to visual or smell impacts in areas that are accessible to people. | Losses to biomass and abundance are likely to be the most important factors in impacting the supply of this service. | Function |
| **R&M: Erosion prevention and sediment retention**  Indirect link *Macroalgae* | Sargassum that washes ashore provides beach stabilisation and erosion prevention by consolidating sediment (Pendleton et al., 2014). | Losses to biomass of Sargassum would impact this service, which is indirectly supported by the neuston in oceanic waters. | Function |
| **R&M: Oxygen production**  Direct link  *Macroalgae, Zooneuston* | The photosynthesis of floating macroalgae such as Sargassum and others contributes to the atmospheric supply of oxygen (Hader and Schafer, 1994). Some neuston species also harbour symbiotic single-celled algae, e.g. *Velella* harbour dinoflagellates *Scripsiella spp.* (Venn et al., 2008); oxygen is thus indirectly supplied via the process of photosynthesis in these algal symbionts. | Losses to biomass and abundance are likely to be the most important factors in impacting the supply of this service. | Function |
| **R&M: Seed and gamete dispersal**  Direct link  *Macroalgae, Zooneuston* | *Halobates*, a marine insect, require floating substrates in the open ocean to lay their eggs on. These substrates include other components of the neuston, such as algae (including Sargassum), mollusc shells and insect carcasses, as well as, feathers, small pieces of driftwood (Cheng, 1985, Mahadik et al., 2019) and even microplastic particles (Goldstein et al., 2012). These substrates, including the biological components of the neuston, are essential to *Halobates* to facilitate their dispersal throughout the ocean. | Losses to biomass and abundance of the neuston could limit the successful dispersal of *Halobates* and any other species that rely on this type of dispersal. | Function, Actual use |
| **R&M: Maintaining nursery populations and habitats**  Direct link  *Macroalgae, Zooneuston* | The neuston provide the *Maintaining nursery population and habitats* (of important migratory and commercial species) in two ways. Firstly, species such as *Vellela* sp. are an important food source for charismatic marine predators, such as turtles (Witherington, 2002, Revelles et al., 2007) and sunfish (Nakamura and Sato, 2014), for migratory birds such as the sooty shearwater, species of storm-petrel, shearwater (Ribic et al., 1997), Phalaropes (DiGiacomo et al., 2002) and for commercially important fish species such as tuna (Thiebot and McInnes, 2019, D’Ambra et al., 2015). Although not especially nutritious, it is believed they may have essential properties for nutrition of species that consume them (Thiebot and McInnes, 2019). In addition, when *Vellela* sp. are stranded inshore, they are also known to provide nutrition to shore species including birds and crabs (Purcell et al., 2012, Phillips et al., 2017).  Secondly, the neuston provide a pelagic refugia and substrate, thus physically creating habitat in the open ocean (Doyle et al., 2014, Lynam and Brierley, 2007). Juvenile fish, both pelagic and deep sea benthic, aggregate around neuston and are thought to benefit from both associated shelter and food. Neuston can act as fish aggregating objects and enhance productivity in their surroundings (Thiel and Gutow, 2005). Larger neuston also act as a substrate for a variety of organisms, for example the megalopae of commercially important crab species, which is transported on *Velella* sp (Purcell et al., 2012). Floating clumps of seaweed also provides important habitat for juvenile fish species (Vandendriessche et al., 2007), support more abundant and biodiverse assemblages of fish (Casazza and Ross, 2008) and support juvenile turtle populations (Witherington, 2002) | Losses to biomass and abundance are likely to be the most important factors in preventing the benefits of this service to commercially important and migratory species. | Function, Actual use |
| **R&M: Gene pool protection**  Direct link  *Macroalgae, Zooneuston* | This service involves the protection of genes and species in nature. All species contribute to this service to protect genetic diversity for future generations and genetic insurance. The neuston contribute to this directly through their own genetic diversity, including the presence of endemic species. For example, the *Sargassum* fish (*Histrio histrio*) is a species of frog fish that spends its entire life associated with *Sargassum* beds. The species has also evolved to have appendages that resemble the *Sargassum* seaweed (McEachran, 2015). In addition, the neuston contribute indirectly to gene pool protection through their role as predators, the neuston act as biodiversity regulators, contributing to the enhancement of biodiversity (Doyle et al., 2014). | A loss of biodiversity and an unbalanced food web would contribute to a decline in this service. | Function |
| **R&M: Pest Control**  Direct link  *Macroalgae, Zooneuston* | This service is carried out through the maintenance of a balance of organisms in the ecosystem and a healthy food web. Thus, the neuston carry out this service through the predator-prey interactions that occur within the community, potentially preventing any one species proliferating and causing a pest related problem elsewhere. For example, through their role as predators, the neuston act as biodiversity regulators (Doyle et al., 2014). | A loss of biodiversity and an unbalanced food web would contribute to a decline in this service. | Function |
| **R&M: Disease Control**  Direct link  *Macroalgae, Zooneuston* | This service is carried out through the maintenance of a balance of organisms in the ecosystem and a healthy food web. Thus, the neuston carry out this service through the predator-prey interactions that occur within the community, potentially preventing any one species proliferating and causing a health-related problem elsewhere. There are examples of neustonic organisms, causing problems in e.g. *Physalia* sp. in tourist bathing sites, and these in turn are controlled by their neustonic predators *Janthina* sp. and *Halobates* sp. (Bieri, 1966, Cheng, 1985). | A loss of biodiversity and an unbalanced food web would contribute to a decline in this service. | Function |
| **R&M: Sediment nutrient cycling**  Indirect link  *Macroalgae, Zooneuston* | Neuston contribute to sediment nutrient cycling because they can capture nutrients both from the microbial loop, using symbiotic zooxanthellae, phytoplankton and smaller zooplankton (Doyle et al., 2014, Purcell et al., 2012). When they die or defaecate, their bodies or faeces can sink, with some eventually reaching the seabed, thus contributing to benthic-pelagic coupling and the addition of nutrients from the upper pelagic zone to the sediment. For example, Salps have high rates of filter feeding, retaining a range of particle sizes (< 1 um to 1 mm) and produce large and rapidly sinking faecal pellets as compared with phytoplankton derived particles that sink passively and can take a month or longer to be sequestered in the deep ocean (Graham et al., 2014, and references therein.) Similarly, large amounts of Sargassum seaweed sink to the deep sea bed, providing a considerable input of organic material (Baker et al., 2018). In addition, neuston, such as *Velella*, can get washed ashore in mass strandings, providing a substantial contribution of pelagic derived production to terrestrial and coastal ecosystems, where they are grazed on and contribute to nutrient cycling there (Purcell et al., 2012, Betti et al., 2017). Furthermore, because neuston are preyed on by higher trophic organisms such as turtles, birds and whales, they further contribute to overall nutrient cycling. | A loss of abundance and biomass are likely to impact the supply of this service. This service is indirect because the service is directly supplied in different habitats (benthic, sediment habitats) and not in the open ocean. | Function |
| **R&M: Chemical Condition of Seawater**  Direct link  *Macroalgae, Zooneuston* | Similar to the service above, the neuston are also contributing to the chemical condition of seawater, through getting nutrition from symbiotic zooxanthellae, microbes, phytoplankton and smaller zooplankton, defaecating and dying. Production is also thought to be higher around floating items, such as aggregations of neuston, than in surrounding areas (Thiel and Gutow, 2005). Studies have also shown the concentration of ocean metals including cadmium and lead is different in the neuston than in other pelagic organisms and that they play a role in metal biogeochemical cycling in the oceans (Schulz-Baldes, 1992). Thus, they are altering the ocean chemistry in their surrounding environment. The movement of neuston from the surface to lower waters and back (diel migration), may also play a significant role in biogenic mixing and the transfer of nutrients between pelagic layers (Doyle et al., 2014). | A loss of abundance and biomass are likely to impact the supply of this service. | Function |
| **R&M: Global Climate Regulation**  Direct link  *Macroalgae, Zooneuston* | Because the neuston are at the surface of the water and can consume microbes and phytoplankton and use symbiotic zooxanthellae, they thus convert carbon to a form that can be consumed by higher organisms (Doyle et al., 2014), that may then be stored in body tissues of long lived animals, such as whales. Neuston can also die and sink in substantial quantities resulting in a transfer of carbon to the seabed where it can remain (Sweetman and Chapman, 2015). For example, salps have high rates of filter feeding, retaining a range of particle sizes (< 1 um to 1 mm) and produce large and rapidly sinking faecal pellets as compared with phytoplankton derived particles that sink passively and can take a month or longer to be sequestered in the deep ocean (Graham et al., 2014, and references therein). Similarly, large amounts of Sargassum seaweed sink to the deep sea bed, providing a considerable input of organic material (Baker et al., 2018). In addition, the presence of neuston in the upper layer of the ocean also results in greater respiration that can influence the exchange of carbon dioxide at the air-sea interface (Dandonneau et al., 2008). | A loss of abundance and biomass are likely to impact the supply of this service. | Function |
| **C: Recreation & Leisure**  Indirect link  *Macroalgae, Zooneuston* | Neuston provide opportunities for recreation and leisure when they wash up on shore and are observed by beach combers and other shoreline users (Viney, 2014). Additionally, the neuston contains juveniles of a number of species that can be important to contributing to recreational activities such as fishing e.g. Salmon (Pool et al., 2012). | A loss in abundance and species richness is likely to impact this service. Because the neuston are not collected and/or used for recreation in oceanic waters but in another habitat, this service has an indirect link. That is, the neuston in oceanic waters, directly contribute to this service in other habitats. | Actual use |
| **C: Scientific**  Direct link  *Macroalgae, Zooneuston* | Neuston provide this service when they are the subject matter of scientific research activities in the field (in-situ) or ex-situ, for example Purcell et al. (2012). Methods are being developed to optimise the use and viability of macroalgae, such as *Sargassum*, as a source of biofuel (Jones et al., 2020). Automated detection methods where modern artificial intelligence-based approaches are being developed to automatically detect the quantity and type of jellyfish in the Mediterranean Sea (Martin-Abadal et al., 2020); not only would methods like this aid the monitoring of the neuston; it also encourages citizen science projects. | A loss of biodiversity, abundance and biomass are likely to have a negative impact on the supply of this ecosystem service. Losses to species that we do not know of yet, or that we know little about is particularly pertinent to this service, as we will lose any future potential scientific value. | Actual (current use) and Potential (such as, new species or discoveries) use |
| **C: Educational**  Direct link  *Macroalgae, Zooneuston* | Neuston provide this service when they are used as educational subject matter, in-situ or ex-situ (e.g. in the classroom, a laboratory). | The main impact on the service is likely to be from a loss of biodiversity, but depending on the specific educational activity, a loss in abundance and biomass might also have an impact (for example if the activity was practical). Losses to species that we do not know of yet, or that we know little about is particularly pertinent to this service, as we will lose any future potential educational value. | Actual (current use) and Potential (such as, new species) use |
| **C: Heritage**  Direct link  *Macroalgae, Zooneuston* | The names of some of the components of the neuston are associated with cultural heritage, for example the Portuguese-Man-of-War, named after a British Royal Navy expression for war ships in the 16^th^-19^th^ century (Wikipedia, 2020b); the by-the-wind-sailor (Velella velella), which, due to washing up in such high abundances on shores, may have a cultural significance for some people (Viney, 2014). In Italy the common name for Velella velella is 'the small boat of Saint Peter' ('la barchetta di San Pietro'). This is associated with rural cultural heritage and tradition in the north of Italy, with the presence of the *Velella* forecasting a good harvest (Sea-Nature-Studies, 2016). *Glaucus* sp., is named after the Greek sea god (Wikipedia, 2020a). Components of the neuston can therefore form part of our enhance heritage and cultural identity.  Eels, that which migrate to the open ocean and form part of the neuston, have significance in many cultures. For example, European eels are part of English heritage, with jellied eels being a dish that originated in the East End of London in the 18^th^ century, supporting regional economies as far as Norfolk (Norfolk Coast Partnership, 2020). More recently, the popularity of jellied eels have increased again as shoppers seek inexpensive and nutritious foods (Dorward and Noss, 2013). Eels were also used to pay for rent or fishing rights and their dried skin was plaited into a ring and used for wedding bands and chastity belts (Norfolk Coast Partnership, 2020). Eel ‘babbing’ competitions were held on Blakeney Marshes in Norfolk, England, with people catching eels with a baited thread of wool (Norfolk Coast Partnership, 2020).  For extensive examples of the cultural services supplied by macroalgae (including *Sargasssum*) see Pérez-Lloréns et al. (2020) who summarised folklores, mythologies and poems inspired by macroalgae across cultures. Oceanic *Sargassum* also had a place in maritime history, with Christopher Columbus credited for having left a detailed account of the seaweed of the Sargasso Sea on his voyage across the Atlantic in 1492, which have subsequently led to the Sargasso Sea featuring prominently in nautical lore, where sailors believed and feared that their ships would become entangled by seaweed (Ryther, 1956). Portuguese sailors were also credited for the naming of Sargasso sea, as the yellow air bladders on the seaweed reminded them of the grapes “salgazo”or “sargaco” in Portuguese (Ryther, 1956, Online Etymology Dictionary, 2020). | Cultural heritage does not necessarily have a relationship with current ecosystem state, but the persistence of relevant species adds to keeping aspects of cultural heritage alive and in some cases, a loss of abundance in e.g. *Physalia* or *Vellela*, could result in impacting the supply of this service. Thus, this service is given a direct link, though there are both direct and indirect links. | Actual use |
| **C: Entertainment**  Direct link  *Macroalgae, Zooneuston* | Neuston provide this service when they are viewed or experienced for entertainment ex-situ, for example watching a wildlife documentary (that is filmed in-situ). In popular culture, the musician and singer Bjork features a *Glaucus* sp. in her music video ‘Utopia’ (Bjork, 2017), which in this case can be considered as entertainment for the viewers (but can also be considered under aesthetics for the artist, see below). | The main impact on the service is likely to be from a loss of biodiversity, but depending on the context, a loss in abundance and biomass may also have an impact. | Actual and Potential use |
| **C: Aesthetic**  Direct link  *Macroalgae, Zooneuston* | The neuston is a source of artistic inspiration. For example, on the website Etsy, a search for ‘blue dragon sea slug’ yields around 20 artistic products inspired by *Glaucus* sp., while a search for ‘jellyfish’ yields over 15,000 and includes representations of *Physalia* sp; for macroalgae, a search for ‘seaweed pressing’ and ‘Sargassum’ returned 139 and 8 results respectively (Etsy, 2020). In popular culture, the musician and singer Bjork features a *Glaucus* sp. in her music video ‘Utopia’ (Bjork, 2017), which in this case can be considered as the artistic or aesthetic inspiration for the artist who produced the video, but can also be considered as entertainment for the viewers (see above *Entertainment*). A further example is that of artist Aaron Ansarov, who photographs live specimens of *Physalia* sp. (man-of-war) to create artistic images (Davis, 2013). | Inspiration can come from second-hand representations (e.g. photographs) and straight from the source. Losses in species abundance and richness are likely to impact on the supply of this service. Greater species richness offers greater scope for artistic inspiration, while greater abundance offers greater likelihood of people observing the species. Thus, while some inspiration may come from indirect sources, some can come directly, this service is given a direct link. | Actual and Potential use |
| **C: Symbolic**  Direct link  *Macroalgae, Zooneuston* | Components of the neuston can form part of symbology in culture such as through use as symbols or logos for companies or businesses, enhancing their identity. For example, the Australian ‘Bluebottle Brewing’ company, that uses a common name and images of *Physalia* sp. to represent their brand (DesignCrowd, 2019). | Symbolism does not necessarily have a relationship with current ecosystem state, but the persistence of relevant species adds to keeping aspects of symbolism alive and supply of the service may be impacted by loss of important species. Thus, this service is given a direct link. | Actual use |
| **C: Sacred and/or Religious**  Indirect link *Macroalgae,*  *Zooneuston* | Components of the neuston can form part of or enhance spiritual identity in some cultures. For example, in Italy the common name for Velella velella is 'the small boat of Saint Peter' ('la barchetta di San Pietro'). This is associated with rural tradition in the north of Italy, with the presence of the *Velella* forecasting a good harvest (Sea-Nature-Studies, 2016). This example has both religious and sacred connotations. A further example connecting the neuston to the sacred is that of the neustonic snail *Janthina*, which has been investigated as the *hillazon*, a marine animal source of a sacred Jewish blue dye, *tekhelet*, as suggested by The Talmud. Although the source is now believed to be a benthic snail species (Wikipedia, 2020c).  Eels often appear in sacred contexts in different cultures, for example, in England, their skin was plaited into a ring and used for wedding bands and chastity belts (Norfolk Coast Partnership, 2020), demonstrating their significance in this context (see also Heritage). See Pérez-Lloréns et al. (2020) who summarised the sacred associations of macroalgae in different cultures. These could include floating macroalgae. | Sacred/Religious aspects do not necessarily have a relationship with current ecosystem state, but supply of the service may be impacted by loss of important species. We assign this service an indirect link because the service is associated with particular practices or location specific beliefs and the contribution to these is likely to be supplied in areas assessible to people, such as coastal areas, and not directly in the open ocean. | Actual use |
| **C: Existence**  Direct link  *Macroalgae, Zooneuston* | Biodiversity of the neuston intrinsically has value simply by virtue of its existence where people appreciate that it is there, even if they never have any use for it. | The impact on this service is from a loss of biodiversity. | Function |
| **C: Bequest**  Direct link  *Macroalgae, Zooneuston* | Biodiversity of the neuston has bequest value when people want to protect its existence for future generations, a moral/ethical perspective, or belief. This can be carried out actively through protecting species or habitats in some way. | The impact on this service be from a loss of biodiversity. | Function |

^1^Marine ecosystem services not supplied by the neuston include: Plant and algal seafood from aquaculture; Animal seafood from aquaculture; Plant and algal-based biofuels; Animal-based biofuels; Flood protection.

**Table S2** Summary matrix of ecosystem services (ecological and societal benefits of neuston) provided by the zooneuston and macroalgae found on the ocean surface i.e. the neuston. There are three types of service: Provisioning, Regulation and maintenance, and Cultural. Definitions of the services are derived from (Culhane et al., 2019). Links can be direct or indirect (see methods in main text), for further explanations of links see Table S1.

References

ATWELL, C. 2011. *Mulch to gain from Sargassum* [Online]. Nation News. Available: <https://www.nationnews.com/nationnews/news/1816/mulch-gain-sargassum> [Accessed 29 July 2020].

BAKER, P., MINZLAFF, U., SCHOENLE, A., SCHWABE, E., HOHLFELD, M., JEUCK, A., BRENKE, N., PRAUSSE, D., ROTHENBECK, M., BRIX, S., FRUTOS, I., JÖRGER, K. M., NEUSSER, T. P., KOPPELMANN, R., DEVEY, C., BRANDT, A. & ARNDT, H. 2018. Potential contribution of surface-dwelling Sargassum algae to deep-sea ecosystems in the southern North Atlantic. *Deep Sea Research Part II: Topical Studies in Oceanography,* 148**,** 21-34.

BETTI, F., BAVESTRELLO, G., BO, M., COPPARI, M., ENRICHETTI, F., MANUELE, M. & CATTANEO-VIETTI, R. 2017. Exceptional strandings of the purple snail Janthina pallida Thompson, 1840 (Gastropoda: Epitoniidae) and first record of an alien goose barnacle along the Ligurian coast (western Mediterranean Sea). *The European Zoological Journal,* 84**,** 488-495.

BIERI, R. 1966. Feeding preferences and rates of the snail,  *Ianthina prolongata*, the barnacle, *Lepas anserifera*, the nudibranchs, *Glaucus atlanticus* and *Fiona Pinnata*, and the food web in the marine neuston. *Publications of the Seto Marine Biological Laboratory,* 14**,** 161-170.

BJORK. 2017. *Utopia* [Online]. YouTube. Available: <https://www.youtube.com/watch?v=Sqbv7cCM5AI> [Accessed 10 February 2020].

BREKHMAN, I. I., GOLOTIN, V. G., GONENKO, V. A., MOLOKOVA, L. P. & KHASINA, E. I. 1981. Antioxidant activity of neuston and plankton extracts from vostok bay, sea of Japan. *Comparative Biochemistry and Physiology Part B: Comparative Biochemistry,* 70**,** 381-383.

BUCKMAN, K. L., LANE, O., KOTNIK, J., BRATKIC, A., SPROVIERI, F., HORVAT, M., PIRRONE, N., EVERS, D. C. & CHEN, C. Y. 2018. Spatial and taxonomic variation of mercury concentration in low trophic level fauna from the Mediterranean Sea. *Ecotoxicology,* 27**,** 1341-1352.

CARIBBEAN ALLIANCE FOR SUSTAINABLE TOURISM. 2015. *Sargassum: A resource guide for the Caribbean* [Online]. Barbados: Atlantis Submarines. Available: <https://www.barbados.atlantissubmarines.com/upimages/articles/document/1527150332Sargassum-Resource-Guide-For-the-Caribbean.pdf> [Accessed July 27 2020].

CASAZZA, T. & ROSS, S. 2008. Fishes associated with pelagic Sargassum and open water lacking Sargassum in the Gulf Stream off North Carolina. *Fishery Bulletin,* 106.

CECI, L. 1975. Fish Fertilizer: A native North American practice? *Science,* 188**,** 26-30.

CHENG, L. 1985. Biology of *Halobates* (Heteroptera: Gerridae). *Annual Review of Entomology,* 30**,** 111-135.

CULHANE, F., FRID, C. L. J., ROYO-GELABERT, E. & ROBINSON, L. 2019. EU policy-based assessment of the capacity of marine ecosystems to supply ecosystem services. ETC/ICM Technical Report 2/2019: European Topic Centre on Inland, Coastal and Marine Waters, 269 pp.

D’AMBRA, I., GRAHAM, W. M., CARMICHAEL, R. H. & HERNANDEZ, F. J. 2015. Fish rely on scyphozoan hosts as a primary food source: evidence from stable isotope analysis. *Marine Biology,* 162**,** 247-252.

DANDONNEAU, Y., MENKES, C., DUTEIL, O. & GORGUES, T. 2008. Concentration of floating biogenic material in convergence zones. *Journal of Marine Systems,* 69**,** 226-232.

DAVIS, N. 2013. Hidden beauty of the portuguese man o'war. *The Observer*, 12 October.

DEANE, G. 2013. *Sargassum sea vegetatble* [Online]. Eat the Weeds. Available: <http://www.eattheweeds.com/Sargassum-not-just-for-breakfast-any-more-2/> [Accessed 25 July 2020].

DESIGNCROWD. 2019. *Playful, bold, craft brewery logo design for a company in Australia by Rajitoas* [Online]. Available: <https://www.designcrowd.com/design/21088605> [Accessed 14 July 2020].

DIGIACOMO, P. M., HAMNER, W. M., HAMNER, P. P. & CALDEIRA, R. M. A. 2002. Phalaropes feeding at a coastal front in Santa Monica Bay, California. *Journal of Marine Systems,* 37**,** 199-212.

DORWARD, J. & NOSS, A. 2013. Britain falls for the joy of jellied eels. *The Guardian*.

DOYLE, T. K., HAYS, G. C., HARROD, C. & HOUGHTON, J. D. R. 2014. Ecological and Societal Benefits of Jellyfish. *In:* PITT, K. A. & LUCAS, C. H. (eds.) *Jellyfish Blooms.* Dordrecht: Springer Netherlands.

ETSY. 2020. *Etsy* [Online]. Available: <https://www.etsy.com/> [Accessed 5 February 2020].

EWING-CHOW, D. 2019. *Meet St. Lucia's First Indigenous Biotech Company* [Online]. Frobes. Available: <https://www.forbes.com/sites/daphneewingchow/2019/02/01/meet-the-caribbeans-first-indigenous-biotech-company/#:~:text=Algas%20Organics'%20flagship%20product%2C%20Algas,every%20pound%20of%20Sargassum%20seaweed>. [Accessed July 29 2020].

GOLDSTEIN, M. C., ROSENBERG, M. & CHENG, L. 2012. Increased oceanic microplastic debris enhances oviposition in an endemic pelagic insect. *Biology Letters,* 8**,** 817-820.

GRAHAM, W. M., GELCICH, S., ROBINSON, K. L., DUARTE, C. M., BROTZ, L., PURCELL, J. E., MADIN, L. P., MIANZAN, H., SUTHERLAND, K. R., UYE, S.-I., PITT, K. A., LUCAS, C. H., BØGEBERG, M., BRODEUR, R. D. & CONDON, R. H. 2014. Linking human well-being and jellyfish: ecosystem services, impacts, and societal responses. *Frontiers in Ecology and the Environment,* 12**,** 515-523.

HADER, D. P. & SCHAFER, J. 1994. Photosynthetic oxygen production in macroalgae and phytoplankton under solar irradiation. *Journal of Plant Physiology,* 144**,** 293-299.

HEMPEL, G. & WEIKERT, H. 1972. The neuston of the subtropical and boreal North-eastern Atlantic Ocean. A review. *Marine Biology,* 13**,** 70-88.

HIGUERA-CIAPARA, I., FÉLIX-VALENZUELA, L. & GOYCOOLEA, F. M. 2006. Astaxanthin: A Review of its Chemistry and Applications. *Critical Reviews in Food Science and Nutrition,* 46**,** 185-196.

JONES, E. S., RAIKOVA, S., EBRAHIM, S., PARSONS, S., ALLEN, M. J. & CHUCK, C. J. 2020. Saltwater based fractionation and valorisation of macroalgae. *Journal of Chemical Technology and Biotechnology,* 95**,** 2098-2109.

KRIS-ETHERTON, P. M., HARRIS, W. S. & APPEL, L. J. 2002. Fish Consumption, Fish Oil, Omega-3 Fatty Acids, and Cardiovascular Disease. *Circulation,* 106**,** 2747-2757.

LEÓN, J., TAPIA, G. & AVALOS, R. 2005. Partial purification and characterization of an antimicrobial substance produced by a marine Alteromonas sp. *Revista Peruana de Biologia,* 12**,** 359-368.

LOUIME, C., FORTUNE, J. & GERVAIS, G. 2017. Sargassum invasion of coastal environments: A growing concern. *American Journal of Environmental Sciences,* 13**,** 58-64.

LYCZKOWSKI-SHULTZ, J. & HANISKO, D. 2007. A time series of observations on red snapper larvae from SEAMAP surveys, 1982-2003: Seasonal occurrence, distribution, abundance, and size. *Am. Fish. Soc. Symp.,* 60**,** 3-23.

LYNAM, C. & BRIERLEY, A. 2007. Enhanced survival of 0-group gadoid fish under jellyfish umbrellas. *Marine Biology,* 150**,** 1397-1401.

MAHADIK, G. A., AGUSTI, S. & DUARTE, C. M. 2019. Distribution and Characteristics of Halobates germanus Population in the Red Sea. *Frontiers in Marine Science,* 6.

MARTIN-ABADAL, M., RUIZ-FRAU, A., HINZ, H. & GONZALEZ-CID, Y. 2020. Jellytoring: Real-time jellyfish monitoring based on deep learning object detection. *Sensors,* 20**,** 1708.

MARTINCIÉ, D., NÜRNBERG, H. W., STOEPPLER, M. & BRANICA, M. 1984. Bioaccumulation of heavy metals by bivalves from Lim Fjord (North Adriatic Sea). *Marine Biology,* 81**,** 177-188.

MCCLEAVE, J. D., KLECKNER, R. C. & CASTONGUAY, M. 1987. Reproductive sympatry of American and European eels and implications for migration and taxonomy. *American Fisheries Society Symposium,* 1**,** 286-297.

NAKAMURA, I. & SATO, K. 2014. Ontogenetic shift in foraging habit of ocean sunfish *Mola mola* from dietary and behavioral studies. *Marine Biology,* 161**,** 1263-1273.

NOAA FISHERIES. 2020. *Atlantic Menhaden: Commerical Fishing* [Online]. Available: <https://www.fisheries.noaa.gov/species/atlantic-menhaden#commercial> [Accessed July 29 2020].

NORFOLK COAST PARTNERSHIP. 2020. *The Glaven Eel Project: Background to the project* [Online]. Available: <http://www.norfolkcoastaonb.org.uk/partnership/background-to-the-project/1205> [Accessed July 30 2020].

ONLINE ETYMOLOGY DICTIONARY. 2020. *sargasso* [Online]. Available: <https://www.etymonline.com/word/sargasso#etymonline_v_22740> [Accessed 31 July 2020].

PENDLETON, L., KROWICKI, F., STROSSER, P. & HALLETT-MURDOCH, J. 2014. Assessing the Economic Contribution of Marine and Coastal Ecosystem Services in the Sargasso Sea. NI R 14-05. Durham, NC: Duke University.

PÉREZ-LLORÉNS, J. L., MOURITSEN, O. G., RHATIGAN, P., CORNISH, M. L. & CRITCHLEY, A. T. 2020. Seaweeds in mythology, folklore, poetry, and life. *Journal of Applied Phycology*.

PHILLIPS, N., EAGLING, L., HARROD, C. & REID, N. 2017. Quacks snack on smacks: mallard ducks (Anas platyrhynchos) observed feeding on hydrozoans (Velella velella). *Plankton and Benthos Research,* 12**,** 143-144.

POOL, S. S., REESE, D. C. & BRODEUR, R. D. 2012. Defining marine habitat of juvenile Chinook salmon, Oncorhynchus tshawytscha, and coho salmon, O. kisutch, in the northern California Current System. *Environmental Biology of Fishes,* 93**,** 233-243.

PURCELL, J. E., CLARKIN, E. & DOYLE, T. K. 2012. Foods of Velella velella (Cnidaria: Hydrozoa) in algal rafts and its distribution in Irish seas. *Hydrobiologia,* 690**,** 47-55.

REVELLES, M., CARDONA, L., AGUILAR, A. & FERNÁNDEZ, G. 2007. The diet of pelagic loggerhead sea turtles (Caretta caretta) off the Balearic archipelago (western Mediterranean): Relevance of long-line baits. *Journal of the Marine Biological Association of the United Kingdom,* 87.

RIBIC, C. A., AINLEY, D. G. & SPEAR, L. B. 1997. Seabird associations in Pacific equatorial waters. *Ibis,* 139**,** 482-487.

RYTHER, J. H. 1956. The Sargasso Sea. *Scientific American,* 194**,** 98-108.

SCHULZ-BALDES, M. 1992. Baseline study on Cd, Cu and Pb concentrations in Atlantic neuston organisms. *Marine Biology,* 112**,** 211-222.

SEA-NATURE-STUDIES. 2016. *A mass stranding of Velella velella (Linnaeus, 1758), by-the-wind-sailor, north-east Sicily, April 2015.* [Online]. Available: <https://www.seanature.co.uk/pmnhs_velella.html> [Accessed 14 July 2010].

SUNG, K., KIM, K. S. & PARK, S. 2013. Enhancing degradation of total petroleum hydrocarbons and uptake of heavy metals in a wetland microcosm planted with *Phragmites communis* by humic acids. *International Journal of Phytoremediation,* 15**,** 536-549.

SUSARLA, S., MEDINA, V. F. & MCCUTCHEON, S. C. 2002. Phytoremediation: An ecological solution to organic chemical contamination. *Ecological Engineering,* 18**,** 647-658.

SWEETMAN, A. & CHAPMAN, A. 2015. First assessment of flux rates of jellyfish carcasses (jelly-falls) to the benthos reveals the importance of gelatinous material for biological C-cycling in jellyfish-dominated ecosystems. *Frontiers in Marine Science,* 2.

THIEBOT, J.-B. & MCINNES, J. C. 2019. Why do marine endotherms eat gelatinous prey? *ICES Journal of Marine Science*.

THIEL, M. & GUTOW, L. 2005. The ecology of rafting in the marine environment. II. The rafting organisms and community. *Oceanography and Marine Biology: An Annual Review,* 43**,** 279-418.

VANDENDRIESSCHE, S., MESSIAEN, M., O'FLYNN, S., VINCX, M. & DEGRAER, S. 2007. Hiding and feeding in floating seaweed: Floating seaweed clumps as possible refuges or feeding grounds for fishes. *Estuarine, Coastal and Shelf Science,* 71**,** 691-703.

VENN, A. A., LORAM, J. E. & DOUGLAS, A. E. 2008. Photosynthetic symbioses in animals. *Journal of Experimental Botany,* 59**,** 1069-1080.

VINEY, M. 2014. Another life: By-the-wind sailors bring beach visitors to their knees. *The Irish Times*, 5 July.

WIKIPEDIA. 2020a. *Glaucus* [Online]. Wikipedia. Available: <https://en.wikipedia.org/wiki/Glaucus> [Accessed 11 February 2020].

WIKIPEDIA. 2020b. *Man-of-war* [Online]. Wikipedia. Available: <https://en.wikipedia.org/wiki/Man-of-war> [Accessed 11 February 2020].

WIKIPEDIA. 2020c. *Tekhelet* [Online]. Available: <https://en.wikipedia.org/wiki/Tekhelet> [Accessed 14 July 2020].

WITHERINGTON, B. 2002. Ecology of neonate loggerhead turtles inhabiting lines of downwelling near a Gulf Stream front. *Marine Biology,* 140**,** 843-853.

XIE, E., LIU, D., JIA, C., CHEN, X. L. & YANG, B. 2013. Artificial seed production and cultivation of the edible brown alga *Sargassum naozhouense* Tseng et Lu. *Journal of Applied Phycology,* 25**,** 513-522.

YUCATAN TIMES. 2018. First house entirely made of Sargassum built by Mexican inventor in Quintana Roo. *The Yucatan Times*.

ZAGALSKY, P. F. & HERRING, P. J. 1977. Studies of the blue astaxanthin-proteins of Velella velella (coelenterata: chondrophora). *Philosophical Transactions of the Royal Society B,* 279.
