# Supplementary material for: Estimating the impact of new high seas activities on the environment: the effects of ocean-surface macroplastic removal on sea surface ecosystems
Source: PeerJ. 2023 Apr 27;11:e15021. doi: 10.7717/peerj.15021 (PMC10149055; doi:10.7717/peerj.15021)
Supplement: Supplemental Information 3 [file peerj-11-15021-s003.pdf]

Supporting information (additional model analysis). Estimating the impact of new high seas activities on the environment: The effects of ocean-surface macroplastic removal on sea surface ecosystems

Matthew Spencer[1], Fiona E Culhane[1, 3], Fiona Chong[4, 5], Megan O Powell[7], Rozemarijn J Roland Holst[2], and Rebecca R Helm[6, *]

[1]School of Environmental Sciences, University of Liverpool, UK
[2]Durham Law School, Durham University, UK
[3]School of Biological and Marine Sciences, University of Plymouth, UK
[4]Energy and Environment Institute, University of Hull, UK
[5]Biological and Marine Sciences, University of Hull, UK
[6]The Earth Commons, Georgetown University, US
[7]Department of Mathematics and Statistics, University of North Carolina Asheville, US

January 23, 2023

S1 Model analysis

For clarity, we restate the basic form of the model here. Let n be neuston density (dimensions ML^{-2}), let p be plastic density (dimensions ML^{-2}) and let t be time (dimensions T). We use a logistic population growth model for neuston, coupled with an input-output model for plastic dynamics:

$$\frac{\mathrm{d}n}{\mathrm{d}t} = a_1 n + a_2 n^2 + a_3 np - c_1 kn \tag{S1}$$

$$\frac{\mathrm{d}p}{\mathrm{d}t} = b_1 - b_2 p - c_2 kp. \tag{S2}$$

In the neuston dynamics equation (S1), $a_1 > 0$ denotes neuston proportional population growth rate at low density (dimensions T^{-1}) and $a_2 < 0$ denotes the effect of neuston density on neuston proportional population growth rate (dimensions $\mathrm{M}^{-1}\mathrm{L}^2\mathrm{T}^{-1}$). In the absence of plastic and cleanup the population will increase when rare, and will have carrying capacity $-a_1/a_2$. The parameter a_3 (sign unknown) denotes the effect of plastic on neuston proportional population growth rate (dimensions $\mathrm{M}^{-1}\mathrm{L}^2\mathrm{T}^{-1}$). The positive parameter k denotes the effort devoted to ocean cleanup, measured in some convenient way such as energy, money or area swept per unit time (denoted [effort]T^{-1}), and the positive parameter c_1 denotes the rate of neuston removal per unit effort of cleanup (dimensions [effort]$^{-1}$). We do not include an external input of neuston, as explained in the main text.

In the plastic dynamics equation (S2), the positive parameter b_1 denotes external input of macroplastics into the open ocean (dimensions $\mathrm{ML}^{-2}\mathrm{T}^{-1}$). The positive parameter b_2 denotes the natural loss rate of macroplastics from the layer of the ocean affected by cleanup (dimensions T^{-1}). The positive parameter c_2 denotes the rate of macroplastic removal per unit effort of cleanup (dimensions [effort]$^{-1}$).

S1.1 Equilibrium points

We find the equilibrium values where neuston exist ($n > 0$) of the system given by Equations (S1) and (S2) by setting both equations equal to zero in the presence of cleanup to be

$$n'_{\text{with}} = \frac{-a_1 - a_3 p'_{\text{with}} + c_1 k}{a_2} = \frac{(b_2 + c_2 k)(c_1 k - a_1) - b_1 a_2 a_3}{a_2 (b_2 + c_2 k)} \tag{S3}$$

and

$$p'_{\text{with}} = \frac{b_1}{b_2 + c_2 k}. \tag{S4}$$

In the absence of cleanup, $k = 0$, therefore the equilibrium values reduce to

$$n'_{\text{without}} = \frac{-a_1 - a_3 b_1/b_2}{a_2}. \tag{S5}$$

and

$$p'_{\text{without}} = \frac{b_1}{b_2}. \tag{S6}$$

S1.2 Stability of equilibrium points

The equilibrium given by Equations (S3) and (S4) is locally stable provided it is feasible. The Jacobian $\mathbf{J}$ of the system with dynamics given by Equations S1 and S2 is

$$\mathbf{J} = \begin{pmatrix} a_1 + 2a_2 n + a_3 p - c_1 k & a_3 n \\ 0 & -b_2 - c_2 k \end{pmatrix}.$$

Using the Routh-Hurwitz criteria, the equilibrium above is locally stable provided that $j_{11} + j_{22} < 0$ and $j_{11} j_{22} - j_{12} j_{21} > 0$ (Otto and Day, 2007, pp. 309-312) . The second of these conditions simplifies to $j_{11} j_{22} > 0$, since $j_{21} = 0$. The parameters b_2, c_2 and k are all positive, and so $j_{22} < 0$. Thus the Routh-Hurwitz criteria are satisfied if and only if $j_{11} < 0$. Evaluating at $(n'_{\text{with}}, p'_{\text{with}})$, we obtain $j_{11} = -a_1 - a_3 p'_{\text{with}} + c_2 k$, which is just the numerator of Equation (S3). Since the denominator of Equation (S3) is negative, the Routh-Hurwitz criteria are satisfied and the equilibrium is locally stable if and only if $n'_{\text{with}} > 0$. In other words, the equilibrium is locally stable if and only if it is feasible. A similar analysis shows that if this equilibrium becomes negative, then the equilibrium with $n = 0$ becomes locally stable.

S1.3 Net effect of cleanup

Suppose that the target of cleanup is to reduce the plastic equilibrium to a fraction $1/r$ of its value in the absence of cleanup, where r is a dimensionless number greater than 1. Then

$$\frac{p'_{\text{without}}}{p'_{\text{with}}} = \frac{b_1/(b_2 + c_2 k)}{b_1/b_2} = \frac{1}{r}$$

$$\implies c_2 k = (r-1) b_2.$$

We find the net effect of such a cleanup effort on neuston by find the difference in equilibrium values of the neuston with and without plastic cleanup. Using Equations (S3) and (S5), we have

$$n'_{\text{with}} - n'_{\text{without}} = \frac{-a_1 - a_3 p'_{\text{with}} + c_1 k}{a_2} - \frac{-a_1 - a_3 b_1/b_2}{a_2}$$

Writing

$$p'_{\text{with}} = \frac{b_1}{b_2 + c_2 k} = \frac{b_1}{b_2 + (r-1) b_2} = \frac{b_1}{r b_2},$$

we have

$$\begin{aligned} n'_{\text{with}} - n'_{\text{without}} &= \frac{-a_1 - a_3 b_1/(r b_2) a_2 + c_1 k}{a_2} - \frac{-a_1 - a_3 b_1/b_2}{a_2} \\ &= \frac{-a_1 - a_3 b_1/(r b_2)}{a_2} + \frac{c_1 (r-1) b_2}{a_2 c_2} - \frac{-a_1 - a_3 b_1/b_2}{a_2} \\ &= \frac{c_1 (r-1) b_2}{a_2 c_2} - \frac{a_3}{a_2}\left(\frac{b_1}{r b_2} - \frac{b_1}{b_2}\right) \\ &= \frac{c_1 (r-1) b_2}{a_2 c_2} + \frac{a_3 b_1 (r-1)}{a_2 b_2 r}. \end{aligned}$$

If cleanup has a net positive effect on neuston, then $n'_{\text{with}} - n'_{\text{without}}$ will be positive which implies

$$\begin{aligned} &\frac{c_1 (r-1) b_2}{a_2 c_2} + \frac{a_3 b_1 (r-1)}{a_2 b_2 r} > 0 \\ &\implies \frac{b_2 c_1}{c_2} + \frac{a_3 b_1}{b_2 r} < 0 \quad \text{(dividing by } (r-1)/a_2 \text{ and recalling that } r > 1, a_2 < 0) \\ &\implies \frac{b_2 c_1}{c_2} < -\frac{a_3 b_1}{b_2 r}. \end{aligned} \tag{S7}$$

If Inequality (S7) is reversed, the proposed level of cleanup will have a net negative effect on neuston. Recall b_2, c_1, and c_2 are non-negative parameters, therefore if plastic has no or positive effect on neuston ($a_3 \geq 0$), the inequality cannot be satisfied, implying a negative effect on neuston at any cleanup level. If plastic has a negative effect on neuston ($a_3 < 0$), then we can solve

$$\frac{b_2 c_1}{c_2} = \frac{-a_3 b_1}{r b_2}$$
$$\implies \frac{1}{r} = -b_2 \frac{c_1}{c_2} \frac{b_2}{a_3 b_1}. \tag{S8}$$

to find the reduction of plastic equilibrium ($1/r$) such that there is no effect on neuston. Here, the factor c_1/c_2 is the ratio of efficiencies of neuston removal to plastic removal. The smaller this ratio (i.e. the more selectively the cleanup removes plastic), the further plastic can be reduced without having a negative effect on neuston. The factor $b_2/(a_3 b_1)$ is the reciprocal of the proportional effect of plastic on neuston at the equilibrium plastic density in the absence of cleanup. The more plastic at equilibrium in the absence of cleanup, or the larger the negative effect of a unit of plastic on a unit of neuston, the further plastic can be reduced without having a negative effect on neuston. In words, we can reduce plastic to a fraction

$$\frac{\text{plastic natural loss rate} \times \text{efficiency ratio}}{\text{proportional effect of plastic on neuston in absence of cleanup}}$$

without having a net negative effect on neuston, provided that plastic has a negative effect on neuston proportional population growth rate.

S1.4 Nondimensionalized model

We now nondimensionalize the model in order to reduce the number of parameters and learn more about what determines its behavior. In particular, expressing the equilibrium densities of neuston and plastic as fractions of their equilibrium values in the absence of cleanup allows us to quantify the effects of cleanup even without knowing the values of all the original parameters. We also have to nondimensionalize time. We choose to do this by expressing time relative to the natural plastic loss rate b_2. This is a convenient choice because the natural plastic loss rate plays a central role in determining how much cleanup will affect neuston, for a given proportional reduction in plastic density. Nevertheless, other choices would be possible.

Let $n^* = n/n'_{\text{without}}$ denote neuston concentration as a fraction of its equilibrium value in the absence of cleanup, where n'_{without} is given by Equation (S5) and $p^* = p/p'_{\text{without}}$ denote plastic concentration as a fraction of its equilibrium value in the absence of cleanup, where p'_{without} is given by Equation (S6). Let $t^* = t/\tilde{t}$ denote time scaled by the natural plastic loss rate, where $\tilde{t} = 1/b_2$. Then we can rewrite Equations (S1) and (S2) in nondimensionalized form as

$$\frac{\mathrm{d}n^*}{\mathrm{d}t^*} = n^* \left(\Pi_2 + \Pi_3 n^* + \Pi_4 p^*\right)$$
$$\frac{\mathrm{d}p^*}{\mathrm{d}t^*} = 1 - \Pi_1 p^*$$

where dimensionless parameters are

$$\Pi_1 = \frac{b_2 + c_2 k}{b_2}, \tag{S9}$$

the ratio of plastic cleanup rate plus natural plastic loss rate to natural plastic loss rate,

$$\Pi_2 = \frac{a_1 - c_1 k}{b_2}, \tag{S10}$$

the sum of maximum proportional population growth rate and cleanup effect on neuston, scaled by natural plastic loss rate,

$$\Pi_3 = -\left(\frac{a_1}{b_2} + \frac{a_3 b_1}{b_2^2}\right), \tag{S11}$$

which is -1 times the sum of the maximum proportional population growth rate of neuston scaled by natural plastic loss rate and the proportional effect of plastic on neuston at equilibrium in the absence of cleanup, scaled by natural plastic loss rate, and

$$\Pi_4 = \frac{a_3 b_1}{b_2^2}, \tag{S12}$$

the proportional effect of plastic on neuston at equilibrium in the absence of cleanup, scaled by natural plastic loss rate. Note that the density dependence parameter a_2 does not appear in any of the dimensionless parameters.

The equilibrium of the nondimensionalized systems is

$$p^* = \frac{1}{\Pi_1},$$
$$n^* = \max\left\{0, \frac{-\Pi_2 - \Pi_4/\Pi_1}{\Pi_3}\right\}. \tag{S13}$$

S1.5 Relationship between equilibrium scaled plastic and neuston densities under cleanup

We now make the simplifying assumption that plastic has no effect on neuston proportional population growth rate (i.e. $a_3 = 0$) and study the relationship between scaled plastic and neuston densities at equilibrium. We treat scaled plastic density as under our control, through some management strategy that determines cleanup effort, and examine how this will affect neuston.

Under the assumption of no plastic effect on neuston, $\Pi_4 = 0$ (from Equation S12), Π_3 simplifies to $-a_1/b_2$ (from Equation S11), and from Equations S9, S10 and S13 we can write the scaled equilibrium neuston density as a function of scaled equilibrium plastic density:

$$\begin{aligned} n^*(p^*) &= \max\left\{0, -\frac{\Pi_2}{\Pi_3}\right\} \\ &= \max\left\{0, 1 - \left(\frac{1}{p^*} - 1\right)\Pi\right\}., \end{aligned} \tag{S14}$$

where the dimensionless parameter $\Pi = \frac{b_2}{a_1}\frac{c_1}{c_2}$ is the ratio of natural loss rate of macroplastics to neuston proportional population growth rate at low density, times the ratio of cleanup efficiencies. For a given choice of scaled plastic density p^*, the scaled neuston density depends on the ratio b_2/a_1 of the natural plastic loss rate to the maximum proportional population growth rate of neuston, and the ratio c_1/c_2 of cleanup efficiencies. A neuston population will be most affected if it has slow growth relative to the natural plastic loss rate, and if the cleanup strategy removes neuston at a high rate relative to plastic.

The lowest value to which scaled plastic density can be reduced before the habitat becomes a sink habitat can be found by solving for p^* in Equation (S14) when $n^* = 0$:

$$p^* = \frac{b_2 c_1}{a_1 c_2 + b_2 c_1}.$$

S2 Parameter values

S2.1 Natural loss rate of plastic b_2

Estimates of the natural loss rate of plastic b_2 vary widely, with differences in model assumptions making an important contribution to this variation. We considered the range $0.03\,\mathrm{a}^{-1}$ to $1.26\,\mathrm{a}^{-1}$. The primary mechanism for loss is thought to be fragmentation into microplastics (Koelmans et al., 2017; Lebreton et al., 2019). At the lower end, it has been suggested that the fraction of floating offshore macroplastic fragmenting into microplastic in a year is approximately 0.03 (Lebreton et al., 2019). Under an exponential decay model (the equivalent of this part of their model in continuous time), this gives a lower estimate of

$$b_2 = -\log(1 - 0.03) = 0.03\,\mathrm{a}^{-1}.$$

At the upper end, it has been suggested that under a zero-emission scenario, almost all floating offshore plastic would be lost within 3 a (Koelmans et al., 2017). Unlike our model and that of Lebreton et al. (2019), the fragmentation rate in the Koelmans et al. (2017) model is a quadratic function of plastic mass (their model is in terms of total mass rather than mass density), because of their assumption that this rate depends on surface area. We approximated this by a constant loss rate per unit mass (assumed in our model) over the range of macroplastic masses following the cessation of emissions in their model output. We digitized the portion of the macroplastic curve after emissions ceased in their zero-emission scenario (their Figure 4), and fitted a linear model to the relationship between log plastic mass and time (Figure S1). This underestimates the rate when plastic density is high, and overestimates when plastic density is low, but is the best choice of mean rate for our model, and captures the desired result that almost all macroplastic would be lost within three years. The resulting upper estimate is $b_2 = 1.26\,\mathrm{a}^{-1}$.

S2.2 Neuston proportional population growth rate at low density a_1

There is little information on proportional population growth rates at low density (a_1) for neuston. We therefore used an allometric approach based on body size, which suggested the range $1.08\,\mathrm{a}^{-1}$ to $63.52\,\mathrm{a}^{-1}$ for small neuston species, and the range $0.08\,\mathrm{a}^{-1}$ to $4.75\,\mathrm{a}^{-1}$ for large neuston species. Neuston species likely to be affected by ocean cleanup have approximate body lengths from 1 cm (such as small janthinid gastropods: Lalli and Gilmer, 1989, p. 9) to 30 cm (the length of the pneumatophore of a large Portugese man of war, *Physalia physalis*: Tiralongo et al., 2022). Allometric relationships between maximum proportional population growth rate and body size are typically based on mass rather than length. However, we are not aware of mass:length allometries for invertebrates spanning this length range (for example, those in Peters (1983, Appendix IIa) are for much smaller invertebrates). We therefore assumed that a typical neuston organism is a sphere, and that all such organisms have the same wet mass density, although these are clearly rough approximations. In the absence of data for other species, we estimated this density from data on wet mass per unit area, colony count per unit area and colony length in strandings of *Velella velella* in the North-west Mediterranean (Betti et al., 2019) as

$$\text{wet mass density} = \frac{\text{wet mass per unit area}}{\text{colony count per unit area} \times 4\pi/3 \times (\text{length}/2)^3}.$$

Over six strandings, the range of wet mass densities was $10.59\,\mathrm{kgm}^{-3}$ to $42.77\,\mathrm{kgm}^{-3}$. This is much lower than the density of ocean surface seawater (mean $1025\,\mathrm{kgm}^{-3}$: Koelmans et al., 2017, Table 1), consistent with the observation that many true neuston are positively buoyant (Hempel and Weikert, 1972).

There are few data on maximum proportional population growth rates specifically for invertebrates with such masses, so we obtained estimates from log:log regression using the entire maximum proportional population growth rate and mass data sets in Hatton et al. (2019, a total of 3812 observations). Taking the extremes of the 95% prediction bands from this regression, for masses calculated as above under both low and high wet mass density, gives estimates $a_1 = 1.08\,\mathrm{a}^{-1}$ to $63.52\,\mathrm{a}^{-1}$ for a neuston species with length 1 cm (Figure S2, filled green circles), and $a_1 = 0.08\,\mathrm{a}^{-1}$ to $4.75\,\mathrm{a}^{-1}$ for a neuston species with length 30 cm (Figure S2, open green circles). Differences between the lower and upper bounds of the prediction interval make a much larger contribution to this range than the uncertainty in wet mass density.

S2.3 Efficiency ratio c_1/c_2

Little is known about the efficiency of neuston removal relative to plastic removal (c_1/c_2). Since neuston and floating plastic overlap in size and occur in the same location, 1 is a plausible value for this ratio. However, other values are not implausible, and we therefore considered the range $[1/10, 10]$. Oil booms operate on a similar principle to passive cleanup devices for plastic (and slow-moving active cleanup devices are likely to be little different). Experiments with model oil booms show that the effective depth of the boom can vary by an order of magnitude over wave heights from 0.25 m to 1.25 m, depending on current velocity and wave height (Castro et al., 2010). The concentration of floating microplastics decays approximately exponentially with depth, so that the proportion of such plastic between depths 0 m and d m is $1 - e^{-\lambda d}$, where λ is the decay constant. Field observations suggest that this decay happens more slowly as wave height and wind speed increase (Reisser et al., 2015). At Beaufort number 1, the estimated decay constant for mass concentration is $\lambda = 3.8\,\mathrm{m}^{-1}$, while at Beaufort number 4 (associated with wave heights around 1 m), it is $\lambda = 1.7\,\mathrm{m}^{-1}$ (Reisser et al., 2015). Thus, at Beaufort number 1, a passive device with an effective depth of 3 m could intercept almost all floating microplastic below the part of the ocean surface it spans, while at Beaufort number 4 and an effective depth an order of magnitude less, it might intercept only about 40%. It is thought that the depth distribution of floating macroplastics tends to decay faster with depth than that of microplastics (Reisser et al., 2015). However, neuston such as the hydroid stage of *Velella velella*, which are positively buoyant and have a hydrophobic upper layer, remain at the surface even when when wave height is large (R. Helm, personal observation). Thus, it is possible that almost all neuston might be intercepted under all weather conditions. At Beaufort number 4, an efficiency ratio of $1/0.4 = 2.5$ is therefore quite plausible. However, this is likely to vary among taxa, and would have to be averaged over the weather conditions occurring in the field. Furthermore, the velocity of some neuston taxa relative to a cleanup device may differ from that of floating plastic, because of differences in windage (Egger et al., 2021). Thus, the range $[1/10, 10]$ may be broad, but not implausibly so.

S3 Time scale for effects of cleanup on neuston

Effects of cleanup on neuston density are likely to occur on a time scale of months to decades after the start of a cleanup programme (Figure S3 shows examples where cleanup effort is chosen so that plastic concentration will be halved at equilibrium, and the efficiency ratio of neuston removal to plastic removal is assumed to be 1). For a small neuston species (1 cm length, such as *Janthina globosa*) with the largest plausible proportional

population growth rate at low density (from allometric estimates, as described above), the neuston population will be close to its new equilibrium in less than 1 a (Figure S3, lines with markers). A large neuston species (length 30 cm, such as *Physalia physalis*) with the slowest plausible neuston proportional population growth rates at low density may take several years to approach a new equilibrium (Figure S3, lines without markers), and its concentration will change more slowly if the cleanup effort needed to halve plastic concentration is low (Figure S3, solid line without markers) than if it is high (Figure S3, dashed line without markers).

References

Betti, F., Bo, M., Enrichetti, F., Manuele, M., Cattaneo-Vietti, R., and Bavestrello, G. (2019). Massive strandings of *Velella velella* (Hydrozoa: Anthoathecata: Porpitidae) in the Ligurian Sea (North-Western Mediterranean Sea). *The European Zoological Journal*, 86(1):343–353.

Castro, A., Iglesias, G., Carballo, R., and Fraguela, J. A. (2010). Floating boom performance under waves and currents. *Journal of Hazardous Materials*, 174(1-3):226–235.

Egger, M., Quiros, L., Leone, G., Ferrari, F., Boerger, C. M., and Tishler, M. (2021). Relative abundance of floating plastic debris and neuston in the Eastern North Pacific Ocean. *Frontiers in Marine Science*, 8:626026.

Hatton, I. A., Dobson, A. P., Storch, D., Galbraith, E. D., and Loreau, M. (2019). Linking scaling laws across eukaryotes. *Proceedings of the National Academy of Sciences of the United States of America*, 116(43):21616–21622.

Hempel, G. and Weikert, H. (1972). The neuston of the subtropical and boreal North-eastern Atlantic Ocean. A review. *Marine Biology*, 13:70–88.

Koelmans, A. A., Kooi, M., Law, K. L., and Van Sebille, E. (2017). All is not lost: Deriving a top-down mass budget of plastic at sea. *Environmental Research Letters*, 12(11).

Lalli, C. M. and Gilmer, R. W. (1989). *Pelagic snails: the biology of holoplanktonic gastropod mollusks*. Stanford University Press, Stanford, California.

Lebreton, L., Egger, M., and Slat, B. (2019). A global mass budget for positively buoyant macroplastic debris in the ocean. *Scientific Reports*, 9(1):12922.

Otto, S. P. and Day, T. (2007). *A biologist's guide to mathematical modeling in ecology and evolution*. Princeton University Press, Princeton, New Jersey.

Peters, R. H. (1983). *The ecological implications of body size*. Cambridge University Press, Cambridge.

Reisser, J., Slat, B., Noble, K., Du Plessis, K., Epp, M., Proietti, M., De Sonneville, J., Becker, T., and Pattiaratchi, C. (2015). The vertical distribution of buoyant plastics at sea: An observational study in the North Atlantic Gyre. *Biogeosciences*, 12(4):1249–1256.

Tiralongo, F., Badalamenti, R., Arizza, V., Prieto, L., and Lo Brutto, S. (2022). The Portugese-Man-of-War has always entered the Mediterranean Sea – strandings, sightings, and museum collections. *Frontiers in Marine Science*, 9:856979.

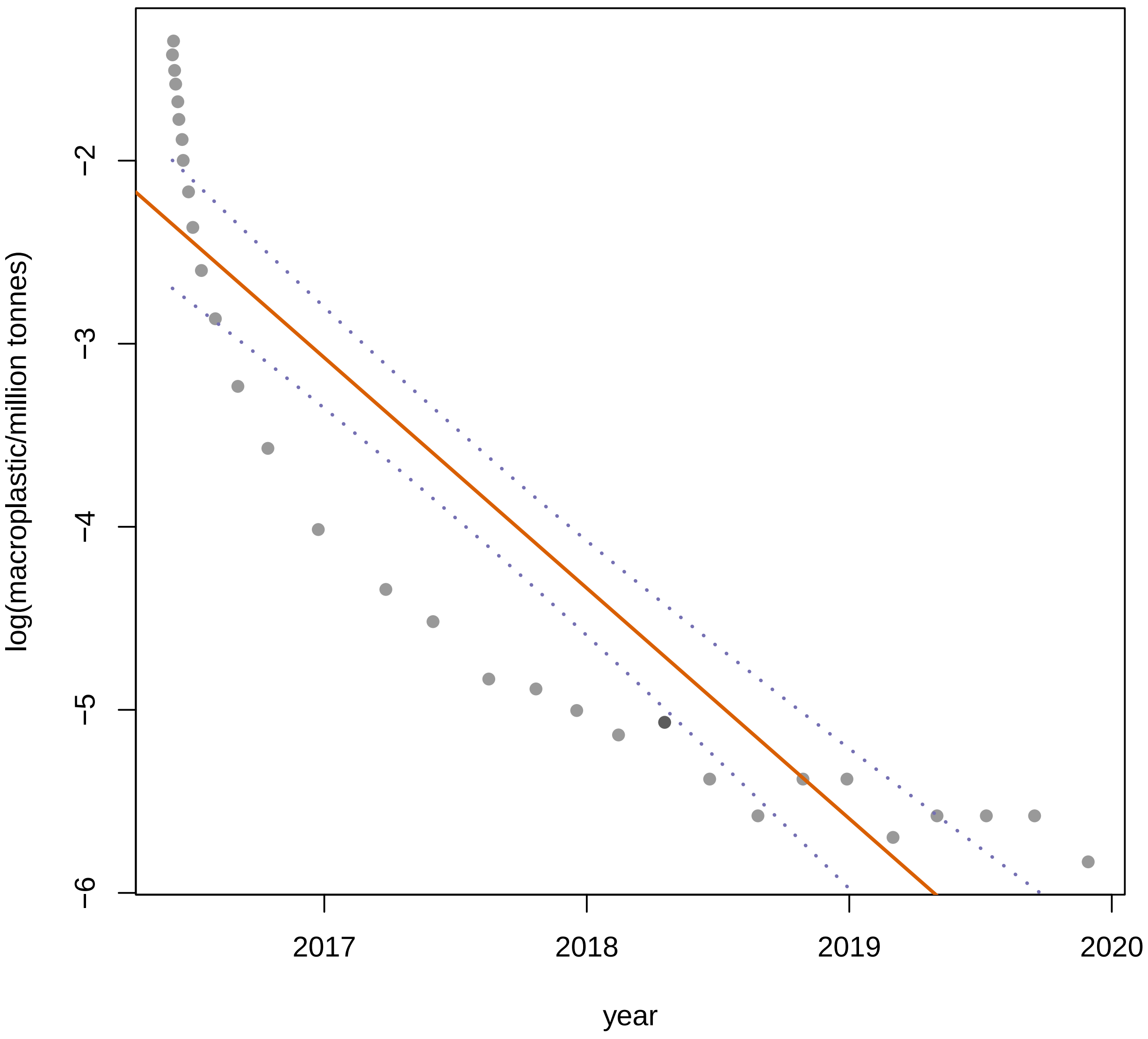

Figure S1: Loss of floating macroplastic over time in the model from Koelmans et al. (2017) (points) and fitted exponential decay model (orange solid line, with purple dotted lines 95% confidence band). Data digitized from Figure 4, macroplastic after cessation of emissions, in Koelmans et al. (2017). Regression model: intercept 2536 (SE 208), slope -1.26 (SE 0.10), residual SE 0.68, $F_{1,30} = 149$, $P < 0.001$, $R^2 = 0.83$.

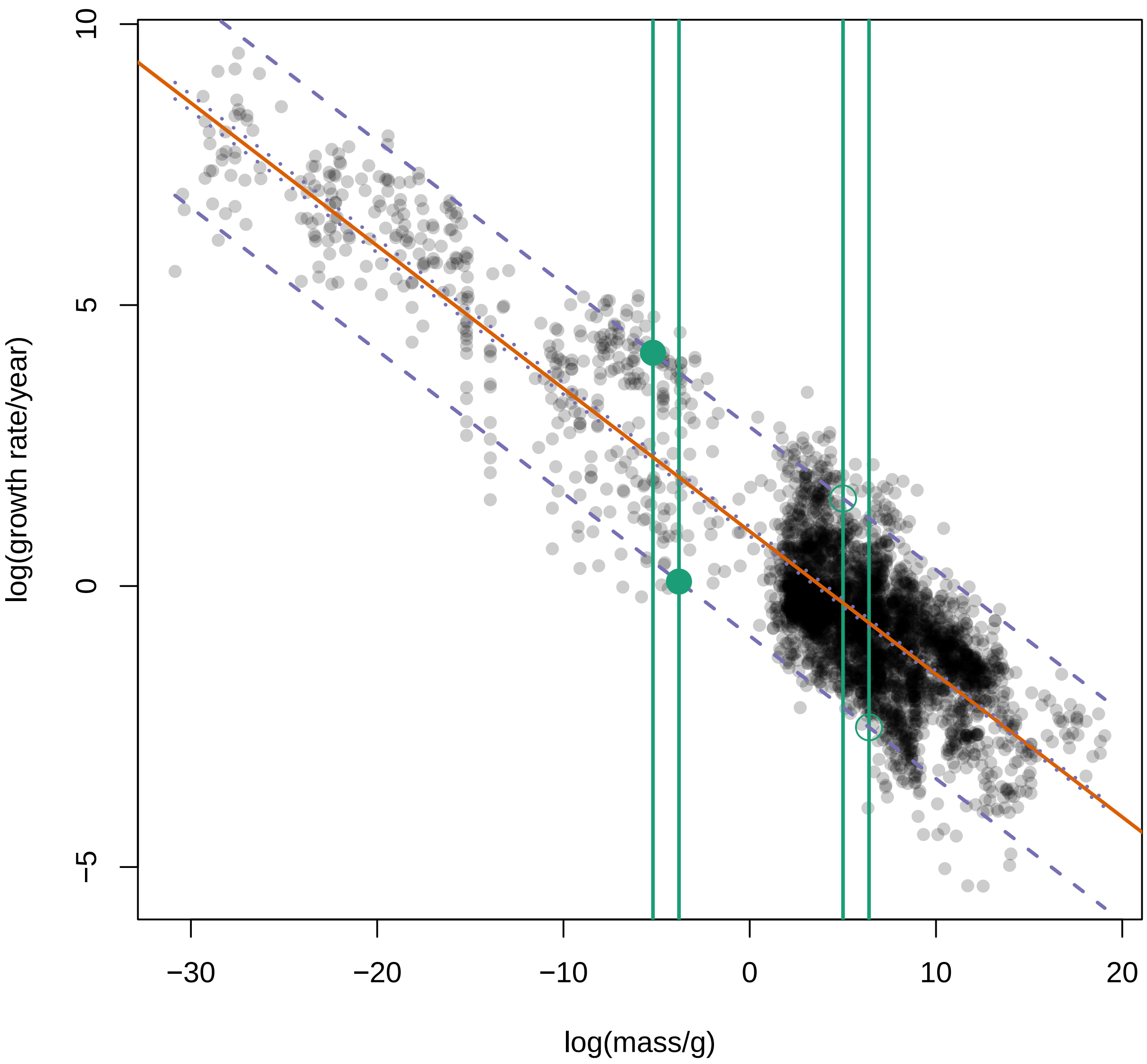

Figure S2: Allometric relationship between the natural log of a_1, the maximum proportional population growth rate per year, and the natural log of wet body mass in grams. Vertical green lines: range of masses for adult neuston taxa likely to be affected by cleanup (in pairs, with low and high values of wet mass density from Betti et al. (2019). Solid orange line: predictions from regression model. Purple dotted lines: 95% confidence band. Purple dashed lines: 95% prediction band. Filled and open green circles: limits of range of natural log of a_1 considered in main text, for small and large neuston respectively. Data from Hatton et al. (2019). Regression model: intercept 0.97 (SE 0.02), slope -0.25 (SE 0.002), residual SE 0.95, $F_{1,3810} = 1.50 \times 10^4$, $P < 0.001$, $R^2 = 0.80$.

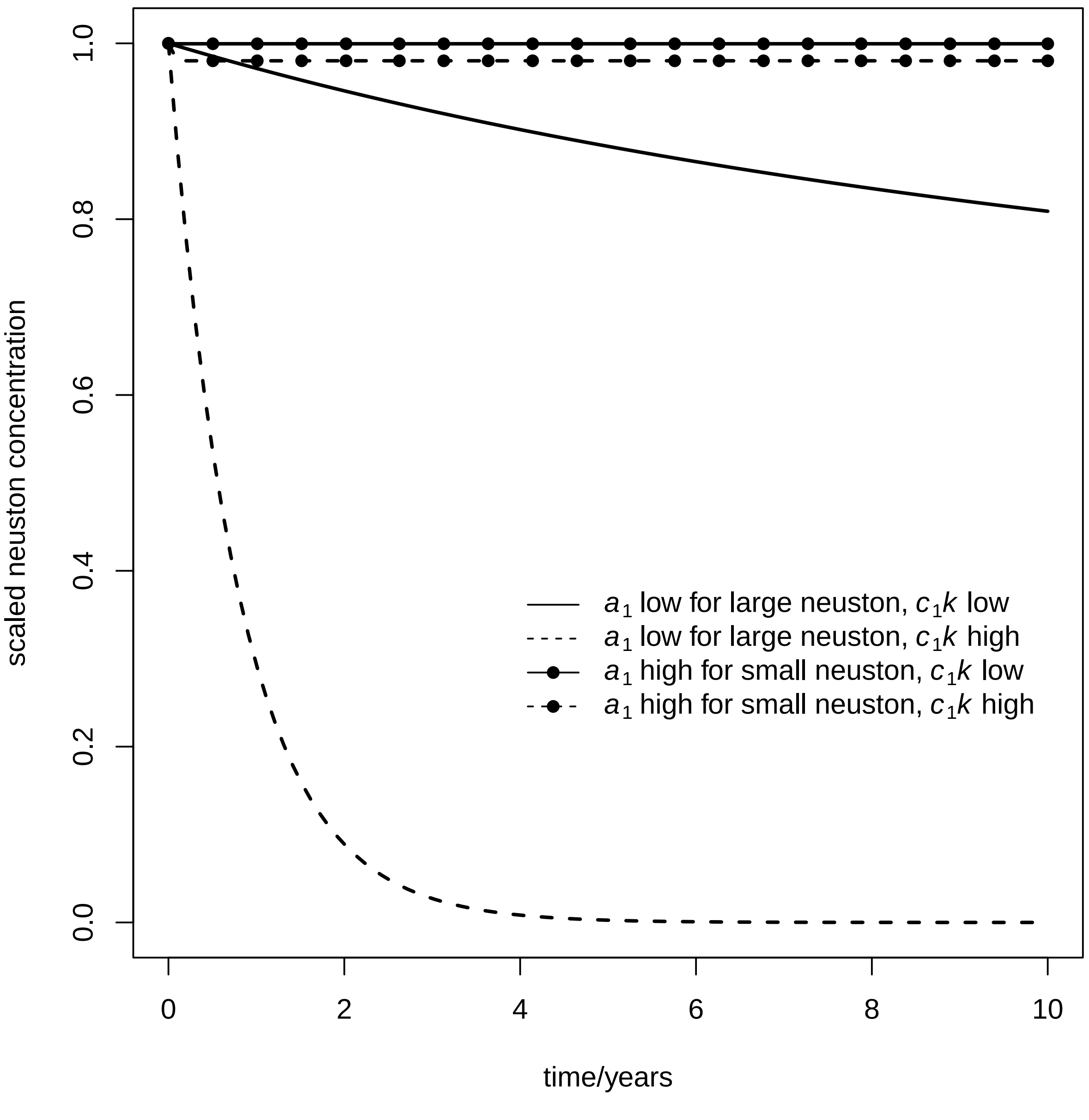

Figure S3: Scaled neuston concentration against time for a range of plausible values of proportional population growth rate at low density a_1 and effect of cleanup on neuston c_1k. The neuston population is scaled relative to its value at equilibrium in the absence of cleanup, and is assumed to be at this equilibrium at time 0. Proportional population growth rate at low density is a low plausible value for a large (30 cm length) neuston species ($0.08\,\mathrm{a}^{-1}$, no markers) or a high plausible value for a small (1 cm length) neuston species ($63.52\,\mathrm{a}^{-1}$, circles). Cleanup effort is chosen so that plastic would be halved ($c_2k = b_2$) at equilibrium under low ($0.03\,\mathrm{a}^{-1}$) or high ($1.26\,\mathrm{a}^{-1}$) natural loss rate of plastic b_2, and the efficiency ratio c_1/c_2 of removal of neuston to plastic is assumed to be 1, so that $c_1k = 0.03\,\mathrm{a}^{-1}$ (solid lines) or $1.26\,\mathrm{a}^{-1}$ (dashed lines).
